# Herbal Medicine Ninjin'yoeito in the Treatment of Sarcopenia and Frailty

**DOI:** 10.3389/fnut.2018.00126

**Published:** 2018-12-12

**Authors:** Nanami Sameshima Uto, Haruka Amitani, Yuta Atobe, Yoshihiro Sameshima, Mika Sakaki, Natasya Rokot, Koji Ataka, Marie Amitani, Akio Inui

**Affiliations:** ^1^Pharmacological Department of Herbal Medicine, Kagoshima University Graduate School of Medical and Dental Sciences, Kagoshima, Japan; ^2^Department of Psychosomatic Internal Medicine, Kagoshima University Graduate School of Medical and Dental Sciences, Kagoshima, Japan; ^3^Education and Research Center for Fermentation Studies, Kagoshima University, Kagoshima, Japan; ^4^Education Center for Doctors in Remote Islands and Rural Areas, Kagoshima University Graduate School of Medical and Dental Science, Kagoshima, Japan

**Keywords:** herbal medicine, kampo medicine, ninjin'yoeito, frailty, sarcopenia, appetite loss, aging, ghrelin-neuropeptide Y signals

## Abstract

Frailty and sarcopenia have recently gained considerable attention in terms of preventive care in Japan, which has an ever-increasing aging population. Sarcopenia is defined as atrophy of skeletal muscles caused by the age-related decrease in growth hormone/insulin-like growth factor and sex hormones. The Japanese Ministry of Health, Labor and Welfare reports that frailty can lead to impairment of both mental and physical functioning. Chronic diseases such as diabetes and dementia may underlie frailty. It is important to prevent progression of frailty and extend the healthy lifespan. In herbal medicine practice, including Japanese Kampo medicine, “Mibyo,” a presymptomatic state, has long been recognized and may be applicable to frailty. Kampo medicines may include several medicinal plants and are thought to have the potential to improve symptoms of frailty, such as loss of appetite and body weight, fatigue, and sarcopenia, as well as anxiety, depression, and cognitive decline. Ninjin'yoeito (Ren Shen Yang Ying Tang) is the most powerful Kampo medicine and has been widely applied to palliative care of cancer patients. This review includes recent anti-aging studies and describes the effects and mechanisms of Ninjin'yoeito (Ren Shen Yang Ying Tang) when used for frailty or to extend a healthy life expectancy.

## Introduction

In Japan, society is aging at an unprecedented rate, substantially changing the social system and disease distribution. Nationwide and community-wide efforts have been made toward ensuring healthy longevity, and paradigm shifts have occurred at various levels. Accordingly, frailty has received attention in preventive medicine practice. The average life span in Japan was reported as 80.98 years in men and 87.14 years in women (Japanese Ministry of Health, Labour and Welfare, 2018). These values continue to increase. The difference between an average life span and healthy life expectancy, namely, the point at which routine daily life becomes limited, is reportedly 8.84 years in men and 12.35 years in women in Japan. These values have remained largely unchanged for a decade. Prevention and treatment of frailty to extend a healthy life expectancy prior to the need for nursing care is a huge challenge in developed societies. In herbal medicine practice, including Kampo medicine in Japan, “Mibyo,” a presymptomatic state, has long been recognized as similarly to frailty. Use of Kampo medicine, especially Ninjin'yoeito (Ren Shen Yang Ying Tang), has been considered for frailty conditions.

### Diagnosis and Pathologies of Frailty

At around 60 years old, we may experience rapid loss of muscle mass and a relative increase in fat mass associated with aging, leading to atrophy of the skeletal muscles (sarcopenia) (1–3). These conditions increase the risk of falls and fractures, requiring long-term care.

**Figure 1:**
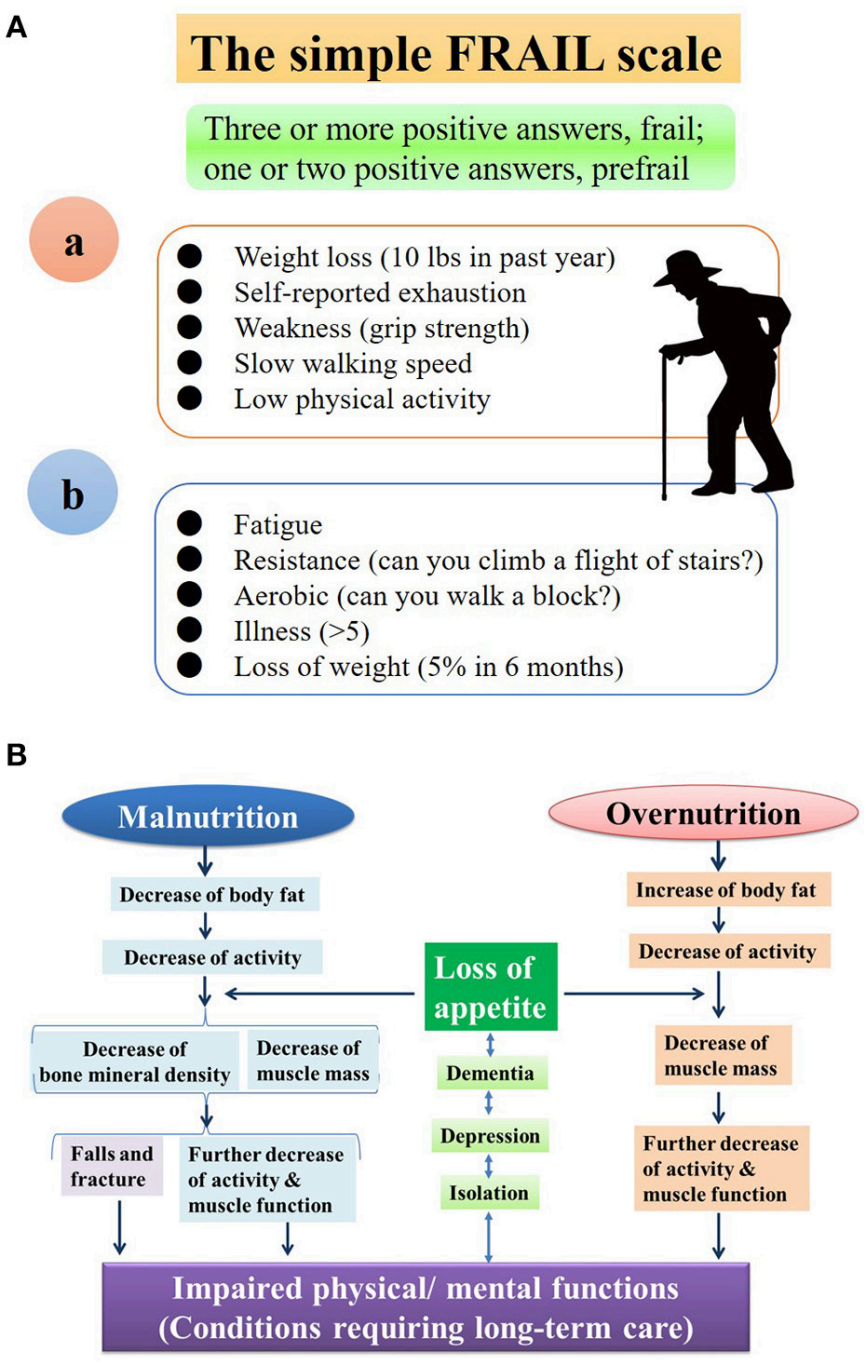
**(A)** Diagnostic Criteria for Frailty by Fried et al. (4) **(a)** and Society on Cachexia and Wasting Disorders **(b)**. In both guidelines, frailty is defined as the presence of at least 3 of 5 criteria, with sarcopenia (atrophy of skeletal muscles) as the basis. Frailty may be close to a presymptomatic state Mibyo in Kampo medicine. The Society on Cachexia and Wasting Disorders **(a)** lists disease aggregation as one of the diagnostic criteria, with frailty cases ranging from mild to severe. Frailty represents a wide range of clinical conditions that encompass emaciation as well as obesity. The appropriate permissions have been obtained from the copyright holders, Sameshima et al. (5). **(B)** Frailty Cascade/Cycle. Either overnutrition or malnutrition can precipitate frailty, in which both physical and psychological vulnerabilities are likely to be seen. Depression and cognitive impairment are either the causes or results of frailty. The presence of depression, for example, not only has a negative effect on treatment, but also worsens sarcopenia by inducing excessive secretion of adrenal cortical hormones or extreme reduction in physical activity. The appropriate permissions have been obtained from the copyright holders, Kuzuya (2).

The Japan Geriatrics Society defined frailty as a state of increased vulnerability in elderly people before the need for long-term care (2014, Figure 1Aa). On the other hand, the Society on Cachexia and Wasting Disorders lists disease progression as one of the diagnostic criteria for frailty (Figure 1Ab), indicating that frailty is more consistent with a syndrome encompassing a variety of physical and mental pathologies, with an emphasis on motor function. The prevalence of frailty is estimated to be about 30% in persons over the age of 80 (2). Frailty can be observed in both malnutrition and overnutrition states and can develop into a vicious cycle known as frailty cycle/cascade, leading to a need for long-term care [(2, 3); Figure 1B]. Physical impairment leads to psychological vulnerability, with depression and cognitive impairment, and vice versa. Depression worsens sarcopenia through excessive secretion of adrenal cortical hormones and/or reduction in physical activity (6–8). Locomotive syndrome is defined as age-related muscle weakness (sarcopenia) and deterioration of motor function due to articular/spinal disease or osteoporosis (4, 9). Although frailty is a psychosomatic pathology and can be divided into physical, social, and cognitive/psychological frailty, locomotive syndrome can be viewed as a clinical condition similarly to physical frailty, with an emphasis on locomotive organs.

**Figure 2:**
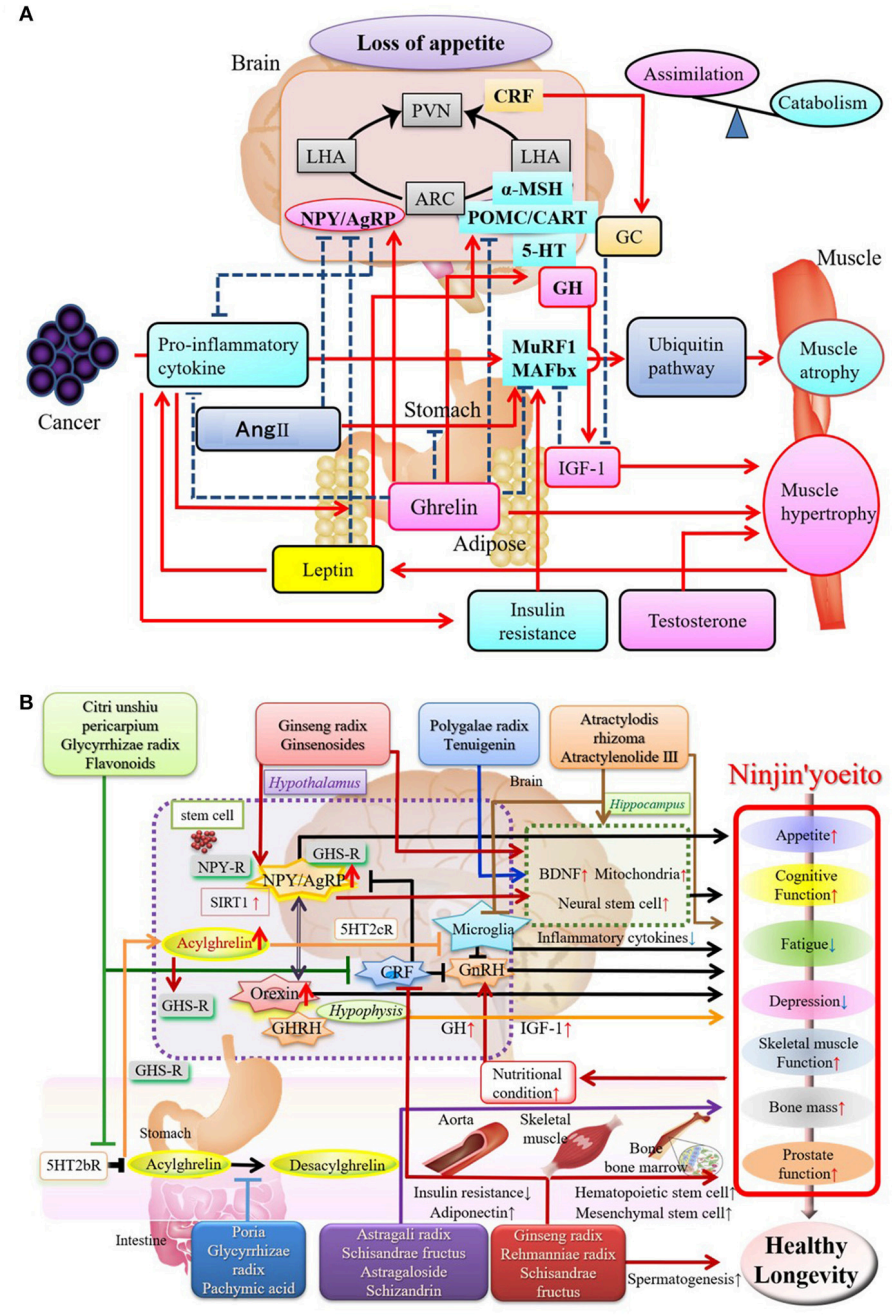
**(A)** Mechanism of Sarcopenia: Positive and Negative Regulators of Skeletal Muscle. Underlying mechanisms of sarcopenia have become increasingly understood through research on brain-gut interactions. Proinflammatory cytokines activate ubiquitin ligases that cause destruction of muscle. The corticotropin-releasing factor (CRF)/glucocorticoid system, insulin resistance, and decreased androgen levels promote sarcopenia, while the hunger hormone, ghrelin, released from the stomach, and insulin-like growth factor (IGF-1) exerts a trophic action on muscle. MuRF1, muscle ring-finger protein 1; MAFbx, muscle atrophy F-box protein (Atrogin-1); IGF-1, insulin-like growth factor 1; Ang II, angiotensin II; NPY, neuropeptide Y; AgRP, agouti-related peptide; POMC, pro-opiomelanocortin; CART, cocaine- and amphetamine-regulated peptide; CRF, corticotrophin-releasing factor; 5-HT, serotonin; PVN, paraventricular hypothalamic nucleus; ARC, arcuate nucleus; LHA, lateral hypothalamic area; HC, glucocorticoids; GH, growth hormone. The appropriate permissions have been obtained from the copyright holders, Amitani et al. (11). **(B)** Components and Active Ingredients of Ninjin'yoeito and their Effects. Many reports have described the role of ginsenosides/saponins from ginseng root on the efficacy of Ninjin'yoeito. Other reported effects include those of ingredients derived from *Atractylodes lancea* rhizome and *Polygala* root on energy metabolism and cognition/emotion. *C. unshiu* peel, *Poria, Glycyrrhiza* root, and panaxadiol derived from ginseng root improve ghrelin signaling underlying the mechanism of action of Ninjin'yoeito, leading to appetite stimulation and improvement in sarcopenia. Ninjin'yoeito stimulates bone marrow hematopoietic and mesenchymal stem cells that may be involved in repair and regeneration of organs and tissues. The appropriate permissions have been obtained from the copyright holders, Inui (3) and Sameshima et al. (5). GHSR, growth hormone secretagogue receptor; NPY-R, NPY receptor; 5HT2cR, 5-HT2c receptor; BDNF, Brain-derived neurotrophic factor; GnRH, Gonadotropin releasing hormone; GHRH, Growth hormone releasing hormone.

Sarcopenia is associated with age-related hormonal changes (decreased growth hormone/insulin-like growth factor [GH/IGF-1] and testosterone) and reduced activity (due to a sedentary lifestyle or osteoarthritis). Cachexia is based on sarcopenia and associated with a variety of diseases that may underlie frailty. Proinflammatory cytokines, including tumor necrosis factor-α, are important in cachexia (10, 11), and may activate the ubiquitin-proteasome system to promote protein catabolism. In contrast, anti-inflammatory cytokines or IGF-1 promote synthesis of muscle proteins or regeneration of muscle fibers. The corticotropin-releasing factor/glucocorticoid system activated by stress or proinflammatory cytokines are other catabolic pathways involving the gut-brain axis [(11); Figure 2A].

### Frailty and Kampo Medicine – With a Focus on Ninjin'yoeito

In the search of PubMed electronic database using the key words: “Ninjin youeito” and “human” or “Ninjin'youeito.” Eighteen and eleven literatures were identified, respectively. We excluded literatures written in Japanese, reviews, animal experiments, and *in vitro* experiments using human cells from the identified literatures. Seven literatures were extracted (Table 1) (12–25).

**Table 1:** Clinical studies of Ninjin'yoeito.

**Citation**	**Participants**	**Symptom**	**Study design**	**Intervention length and measures taken**	**Results**
Hsiao et al. (12)*	Treatment group:	Chronic kidney	Open-label trial	6 months
	*n* = 27, 58.4 ± 13.2 y	Disease		Serum hematocrit and albumin	No change of hematocrit,
	Control group:				Increase in albumin
	*n* = 37, 63.8 ± 14.6 y			Blood inflammatory markers	No change in CRP
				(CRP, IL-6, and TNF-α)	Decrease in IL-6 and TNF-α
				QOL	Improvement in QOL
Xu et al. (13)**	60 (41–81) y: *n* = 33	Non-anemia-related	Open-label trial	6 weeks
		Fatigue with cancer		Patient-reported fatigue rating	Decrease in fatigue severity
Sato et al. (14)	*n* = 5	Healthy	Open-label trial	Single dosage
				Plasma CGRP (calcitonin	Increase in CGRP-IS and
				peptide)-like immunoreactive	sucstance P-IS
				gene-related substances (IS)
				and substance P-IS
Naito et al. (15)	*n* = 5	Healthy	Cross-over	Single dosage
				Plasma motilin,	Increase in motilin,
				vasoactive intestinal peptide (VIP),	gastrin, and somatostatin
				gastrin, and somatostatin	No change in VIP
Cyong et al. (16)	*n* = 34	Hepatitis C virus	Open-label trial	6 months Serum HCV-RNA	Decrease in HCV (8/34)
	*n* = 37	Hepatitis C virus	Open-label trial	3.8 years (7 month- 7 years)
				Viral titer	Viral seroconversion (8/37)
Ito et al. (17)	Lenalidomide with NYT:	Fatigue retrospective study		6 months
	72 (53–85) y, *n* = 13	Multiple myeloma		Fatigue grade	Improvement (12/13) and
					no improvement (1/13) with NYT
	Lenalidomide:				Improvement (11/23) and
	67 (45–79) y, *n* = 23				no improvement (12/23) without NYT
Kudoh et al. (18)	Donepezil with NYT:	Aizheimer's disease	Non-randomized	2 years
	*n* = 12, 74.5 ± 5.4 y	(mild-to-moderate probable)	open-label trial	Mini-mental state ADAS and NIDS,	No change in MMS
	Control group:				Improvement of ADAS and
	*n* = 11, 74.9 ± 3.6 y				NIDS

KCL, Kihon Checklist; CAT, COPD Assessment Test (CAT); HADS, Hospital Anxiety and Depression Scale; ADAS, Alzheimer's Disease Assessment Scale-cognitive component-Japanese version; NIDS, Neuropsychiatric Inventory depression scores; (12)^*^, Prescription is different from that of Japanese (Rehmanniae Radix → Rehmanniae Preparatum Radix, Adding Zizyphi Fructus and Zingiberis Rhizoma); (13)^**^, Prescription is different from that of Japanese(Rehmanniae Radix → Rehmanniae Preparatum Radix, Cinnamon Bark → Cinnamon).

In elderly individuals, polypharmacy is often problematic and may lead to adverse drug reactions (ADRs). Frailty is likely to involve multiple organ systems and may be a good target for multicomponent herbal medicine. Hozai comprises a group of Kampo formulations that restore vitality to patients who have lost psychological and physical energy due to various diseases including cancer. Hozai formulations include Juzentaihoto, Hochuekkito, and Ninjin'yoeito. Kampo theory may regard frailty as Jinkyo, which means dysfunction of Jin, and is associated with production of Ki. Ki is universal energy and a basic element of life in Kampo theory. *Rehmannia* root, a component of Ninjin'yoeito, is often used to treat Jinkyo, which is related to frailty, and is contained in Juzentaihoto and Ninjin'yoeito, but not in Hochuekkito. *Citrus unshiu* peel is contained in Hochuekkito and Ninjin'yoeito, but not in Juzentaihoto. *Polygala* root and *Schisandra* fruit are only contained in Ninjin'yoeito. In cancer palliative medicine, Juzentaihoto or Hochuekkito tend to be prescribed initially, and in serious cases are replaced with Ninjin'yoeito.

Among other crude drugs, ginseng has been used since ancient times. *Panax ginseng* was historically thought to promote immortality, which was sought by the first Qin Emperor. It was imported to Japan in the eighth century, in the era of Emperor Shomu, and has become one of the main components in Ninjin'yoeito. Ninjin'yoeito was frequently used for serious diseases in the Edo Period. The *Heji Jufang*, compiled during the Song Dynasty, states that Ninjin'yoeito is indicated for weakness due to overwork or illness, dullness of the extremities, sharp musculoskeletal pain, shortness of breath, intense low back pain, emptiness and anxiety, thirst and dry mouth, depressive mood, and lethargy, leading to a condition that is difficult to treat. It is also indicated for lung and large intestine symptoms, including cough, sputum production, diarrhea, and vomiting.

In the *Journal of Kampo to Kanyaku*, Domei Yakazu, who was committed to the restoration of Kampo Medicine in the twentieth century, during the Showa period, stated that Ninjin'yoeito can be used for cachexia of cancer, suggesting that it is the most powerful Hozai (3). Ninjin'yoeito is now widely used in the field of palliative medicine, including cancer treatment (3, 26, 27). Ninjin'yoeito increases the rate of remission in advanced gynecological cancer, as assessed by positron emission tomography-computed tomography (26). Ninjin'yoeito is used to prevent toxicity (such as impaired hematopoiesis) associated with anticancer drugs or radiotherapy, and can improve appetite, fatigue, general health status, and even survival. Ninjin'yoeito enhances the therapeutic efficacy of melphalan in multiple myeloma and reduces general malaise (27). Ninjin'yoeito also treats decreased appetite and fatigue in Sjögren's syndrome (28). Many reports on the clinical benefits of Ninjin'yoeito describe improvement of general health status in elderly (29) or postoperative patients (30), amelioration of disordered protein synthesis in hepatic cirrhosis (31) or of diabetic complications such as neuropathy (32), and recovery from anemia (33, 34) or thrombocytopenia (31, 35). In chronic obstructive pulmonary disease (COPD), a major underlying cauthese of cachexia, Ninjin'yoeito treats appetite loss, weight loss, and respiratory symptoms, and improves nutritional status and immune function (36). Ninjin'yoeito, but no other Hozai such as Juzentaihoto and Hochuekkito, treated cough, sputum production, and insomnia. Ninjin'yoeito is effective in control of infection after knee joint replacement (37), and increases bone mineral density in postmenopausal women, treats anosmia resistant to glucocorticoid treatment, and is effective in male infertility. Ninjin'yoeito improves cognitive function and depression in patients with Alzheimer's disease when added to treatment with donepezil (18). There are also many reports suggesting its potential usefulness in home health care and frailty (3).

## Effects of Ninjin'yoeito and Mechanism of Action

Ninjin'yoeito is composed of 12 crude drugs: peony root, Japanese angelica root, C. unshiu peel, Astragalus root, cinnamon bark, ginseng, Atractylodes rhizome, Glycyrrhiza, Rehmannia root, Schisandra fruit, Poria sclerotium, and Polygala root. The main components of this formulation include glycyrrhizic acid, derived from Glycyrrhiza; paeoniflorin from peony root; ginsenosides from ginseng; hesperidin from C. unshiu peel; atractylenolide III from Atractylodes rhizome; isoastragaloside (HQ1/2) from Astragalus root; tenuigenin from Polygala; and schizandrin from Schisandra fruit (Table 2) (38–58). Glycyrrhizic acid has anti-inflammatory effect and has been clinically applied in treatment of chronic hepatic diseases. Paeoniflorin is known to suppress intracellular Ca^2+^ influx and relieves muscle pain. In tumor-bearing animal models treated with anticancer drugs, Ninjin'yoeito not only improves food intake and sarcopenia, but also prolongs survival (59, 60). Ninjin'yoeito may improve the signs of aging and significantly extend survival time in approximately 30% of Klotho-deficient senescence-accelerated mice (59, 60).

**Table 2:** Effective components and indications of crude herbs in Ninjin'yoeito.

**Crude herb**	**Effective component**	**Indication of crude herb**
Ginseng 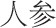	Ginsenosides Rb1, Rg1, Re, Rf, Rd, Rc	Immune enhancement, anti-inflammation effects, antioxidant effects, memory enhancement, platelet-aggregation inhibitory effects, improvement of menopausal disorders, and induction of metabolic energy (38)
Glycyrrhiza 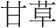	Glycyrrhizin, Liquiritin, Isoliquiritin,	Anti-inflammatory, anti-cancer and immunomodulatory effects (39)
Atractylodes rhizome 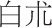	Atractylon, Atractylenolide I, II, III, 3β-acetoxyatractylon, 3β-hydroxyatractylon, Diacetylatractylodiol	Anti-inflammatory and antinociceptive effects (40)
Japanese angelica root 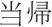	Ligustilide, Butylidenephthalide, Butylphthalide,	Anti-inflammatory effects (41)
Poria sclerotium 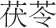	Pachymic acid, Tumulosic acid, Eburicoic acid	Anti-inflammatory anti-apoptotic, and anti-immunologic rejection effects (42)
Cinnamon bark 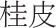	Cinnamic aldehyde, Cinnamyl acetate, Phenylpropyl acetate, Gallic acid	Antidiabetic effects (43), Antihyperglycemic and antihyperlipidemic Action (44)
Polygala root 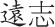	Onjisaponin A, B, E, F, G Tenuifolioside A, B, C, D, E	Antioxidation, anti-inflammation, antidementia, and anti-aging (45, 46)
Citrus unshiu peel 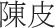	Limonene, Linalool, Terpineol, Hesperidin, Naringin, Poncirin, Nobiletin (-)-synephrine	Antioxidant, anti-inflammatory, antibacterial properties, and anti-cancer activity (47), anti-obesity and lipid-improving effects (48)
Astragalus root 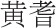	Formononetin, Isomucronulatol, Calycosin, Astragaloside I-Vii, Astragaloside VIII, Astragaloside IV	Anti-aging effect, anti-tumor effects, oxidative stress reduction, immunomodulatory effects, hypolipidemic, antihyperglycemic effects, increase telomerase activity (49)
Schisandra fruit 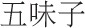	Citral, β-chamigrenal, Citric acid, Malic acid, Tartaric acid, Schizandrin	Anti-aging effect, memory enhancement, enhances myogenic differentiation and inhibiting atrophy, (50–52)
Peony Root 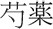	Paeoniflorin, Albiflorin, Oxypaeoniflorin, Benzoylpaeoniflorin, Paeoniflorigenone, Paeonol Phenol β-amyrin	Anti-Inflammatory and immunomodulatory effects (53), stimulate blood circulation and exhibit anti-inflammatory, antiplatelet, and vasodilator activities (54)
Rehmannia Root 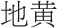	Catalpol, Aucubin, Rehmannioside A, B, C, D, Rehmaionoside A, B, C, Acteoside,	Hypoglycemic effect (55), diuretic effect (55), blood coagulation inhibiting effect (56, 57), immunosuppressive effect (58)

Ginseng, a component of Ninjin'yoeito, shows antifatigue and antidepressant effects in a forced swim test (61). Ginseng may decrease the signs of aging in a senescence-accelerated mouse (SAMP8) (3). Ginsenosides, active compounds from ginseng, are reported to have a wide variety of effects. Ginsenosides ameliorate memory disturbance induced by amyloid beta (62). In a vascular dementia model (middle cerebral artery ischemia/reperfusion), ginsenoside Rg2 improves hemiplegia and memory impairment (63). These results suggest that ginseng has neuroprotective effects. Ginsenoside Rb2 inhibits the decrease in bone mineral density in the femur and 4th lumbar vertebra in ovariectomized mice through the suppression of oxidative stress and osteoclastic cytokines (64). Ginsenoside Rd ameliorates arteriosclerosis and reduces atherosclerotic plaques through inhibition of voltage-independent Ca channels in Apo-E-deficient mice fed a high-fat diet (65). Protopanaxatriol, a metabolite of ginsenoside Rg2, improves insulin resistance (66). Ginsenoside Rg3 suppresses testosterone-induced prostatic hypertrophy and growth of prostate cancer cells through inhibition of mitogen-activated protein kinase signaling (67).

*C. unshiu* peel inhibits amyloid beta-induced neurite atrophy and apoptosis of neural cells. Its components, including hesperidin and narirutin, have been reported to improve cognitive function by promoting reformation of the myelin sheath that is lost during aging (18). Hesperidin treats appetite loss and sarcopenia via suppression of the serotonin pathway and recovery of ghrelin secretion in the stomach [Figure 2A; (68)]. The improvement of sarcopenia by ghrelin can be attributed to the activation of the GH/ IGF-1 system (69).

*Atractylodes* rhizome inhibits cell death by improving mitochondrial activity and intracellular ATP production (70, 71). This protective effect could be important since oxidative stress is considered basic to the pathophysiology of aging (72). Atractylenolide III, a component of *Atractylodes* rhizome, has been reported to ameliorate depression-like symptoms and memory impairment by increasing the expression level of Ca^2+^/calmodulin-dependent protein kinase II and Creb and BDNF in the hippocampus (71).

Adiponectin has been reported to have protective effects on atherosclerosis, and mice with over-expressed adiponectin show prolonged survival, even with a high-fat and high-sucrose diet through inhibition of oxidative DNA damage (73). *Astragalus* root enhances insulin sensitivity via increase of adiponectin, especially its highly-potent high-molecular-weight form (74) and may prevent atherosclerosis.

*Polygala* root and its main component, tenuigenin, promote the growth and differentiation of hippocampal neural stem cells (75). It has been reported to improve cognitive function in adults and elderly subjects in clinical studies (76, 77), and is approved as an over-the-counter drug.

*Schisandra* fruit promotes elimination of fatigue substances, such as lactate and ammonia, from the blood, and increases endurance during exercise on a treadmill via upregulation of peroxisome proliferator-activated receptor γ coactivator 1α, an important factor in skeletal muscle metabolism (78). *Schisandra* fruit increases blood estradiol, uterus estrogen receptor-α and -β, and uterine weight in an ovariectomized post-menopausal model, although it inhibits the proliferation of breast cancer cells (79). Schizandrin is a main component of *Schisandra* fruit.

Ninjin'yoeito is thus expected to reduce physical and psychological vulnerability related to feeding, immunity, emotion, and cognition, which are oftendisturbed in frailty patients (Figure 2B). Ninjin'yoeito could be widely applicable in mild to severe cases of frailty (3).

### Combination Therapy and Adverse Drug Reactions Due to Kampo Medicines

Kampo medicines are composed of a wide variety of crude drugs with pleiotropic effects on the psychosomatic syndrome of frailty, and Ninjin'yoeito is expected to form the basis of these medicines. Recently, Kracie Pharma Ltd. reported special drug use survey results on ADRs associated with Ninjin'yoeito Extract Granules in patients aged ≥65 years (80). The population under analysis consisted of 808 patients (210 males and 598 females, mean age of 77.8 ± 7.35 years; 538 and 262 patients with and without comorbidities; and 664 and 130 taking or not taking concomitant drugs). The incidence of ADRs was 3.09% (25 patients), and gastrointestinal disorders were most common, reported by 17 patients (2.10%). Overall, there were no significant sex-related differences, and approximately 70% of the reported ADRs occurred within 2 months of starting Kampo formulation (80). Given the low and similar incidence of ADRs associated with placebo, the medication should even be safe in the elderly.

In addition, the combined use of Ninjin'yoeito with other Kampo medicines may enhance the effects of therapy. The addition of Yokukansan and Yokukansankachimpihange treats the behavioral and psychological symptoms of dementia (BPSD); Rikkunshito is added for gastrointestinal symptoms, Hangekobokuto for aspiration symptoms, Hachimijiogan/Goshajinkigan for prostate symptoms, and Goshajinkigan for osteoarthritis or spondylosis in severe cases with pain or numbness (81–90). Although Kampo medicines are likely to cause fewer ADRs than modern medicine, multi-combination use requires caution and should be limited to 2 medicines.

## Conclusions

This review describes the clinical application of Kampo medicine in frailty, with a focus on Ninjin'yoeito. As in metabolic syndrome, prevention and treatment of frailty requires diet/exercise, behavioral modification, and utilization of public healthcare resources. Given the progression to a super-aged society, paradigm shifts at both individual and societal levels are needed. The concept of “Mibyo,” a presymptomatic disease state in Kampo medicine, may be a good place to start and frailty could be an important candidate for intervention. It is important to evaluate this presymptomatic state from a scientific perspective to determine how preventive Kampo medicine should be provided. In western medicine Galen is the first to indicate the importance of diet in slowing the aging process (91), and very recently geroprotectors that delay many diseases related to aging are being considered for healthy longevity (92, 93).

Antiaging studies have rapidly evolved, and the mechanisms behind frailty and aging have become increasingly understood. Ninjin'yoeito acts on hematopoietic stem cells to promote the growth and differentiation of erythrocytes, leukocytes, and platelets in animals and humans (3, 94, 95). We recently found that *Polygala* root, *Schisandra* fruit, ginseng, *Rehmannia* root, and *C. unshiu* peel, which are characteristic herbal components of Ninjin'yoeito, promote the growth and differentiation of bone marrow-derived mesenchymal stem cells (66). The components of Ninjin'yoeito may thus be important for their effects on stem cells that may migrate and regulate brain functions associated with feeding and emotion (96–98). Ninjin'yoeito also increases hippocampal neural stem cells (75). These effects on tissue stem cells may underlie the pleiotropic actions on Ninjin'yoeito and suggest its use for frailty.

## Author Contributions

NU wrote the manuscript. NU and AI conceived and organized the structure of the review. NR and KA contributed to the first draft. HA, YA, YS, MS, MA, and AI contributed to critical revision and approved the final manuscript for publication.

### Conflict of Interest Statement

The authors declare that the research was conducted in the absence of any commercial or financial relationships that could be construed as a potential conflict of interest.
